# Normal Values for Speckle-Tracking Echocardiography in Children: A Review, Update, and Guide for Clinical Use of Speckle-Tracking Echocardiography in Pediatric Patients

**DOI:** 10.3390/jcm14041090

**Published:** 2025-02-08

**Authors:** Massimiliano Cantinotti, Guglielmo Capponi, Pietro Marchese, Eliana Franchi, Giuseppe Santoro, Nadia Assanta, Kritika Gowda, Shelby Kutty, Raffaele Giordano

**Affiliations:** 1 Fondazione G. Monasterio CNR-Regione Toscana, Massa, 56124 Pisa, Italy; cantinotti@ftgm.it (M.C.);; 2 Helen B. Taussig Heart Center, Department of Pediatrics, Johns Hopkins Hospital, Baltimore, MD 21205, USA; 3 Advanced Biomedical Sciences, Cardiac Surgery, University of Naples Federico II, Via Pansini, 5, 80131 Naples, Italy

**Keywords:** echocardiography, children, speckle-tracking echocardiography, normal values

## Abstract

**Background/Objectives:** While speckle-tracking echocardiography (STE) is increasingly gaining acceptance in the medical community, establishing normal pediatric values and interpreting data derived from software provided by various vendors can pose significant challenges. This review aims to present an updated compilation of nomograms pertinent to speckle-tracking echocardiography. **Methods**: A review of research using three medical engine searches (National Library of Medicine, Science Direct, and Cochrane Library) for Medical Subject Headings (MeSH) and the free text terms “echocardiography”, “STE”, “normal values”, and ”children” was performed and refined by adding the keywords “nomograms”, “z-scores”, and “healthy children”. **Results**: A total of twenty-five studies were selected for the final analysis. Our research indicated that current nomograms provide adequate coverage of most strain parameters; however, those pertaining to the right ventricle and the atria are less numerous than those for the left ventricle. A noted trend suggests a decrease in strain values with advancing age and increasing body surface area; nevertheless, the relationships observed were weak and nonlinear. The absence of robust correlations between strain values and age and body size parameters hindered the generation of a Z-score possessing sufficient statistical power. Consequently, normal values are primarily represented as mean values accompanied by standard deviation. A comparative analysis of vendors demonstrated good agreement between different versions of the same platform for Philips (except for QLAB 5) and, similarly, between General Electric (GE) and TomTec. The limited data available regarding the comparison between GE and Philips revealed significant findings that warrant further investigation of differences. **Conclusions**: A comprehensive review and an updated list of current pediatric nomograms for STE measurements have been presented. This may serve as a valuable guide for accurately interpreting STE in pediatric patients with congenital and acquired heart disease.

## 1. Introduction

Speckle-tracking echocardiography (STE) is increasingly employed for more precise evaluation of ventricular systolic function in the pediatric population [1,2,3,4]. Atrial strain analysis [5,6] is also receiving heightened attention for assessing acquired and congenital heart defects in children. In recent years, numerous pediatric nomograms regarding major strain parameters have been published [1,2,3,4,5,6,7,8,9,10,11,12,13,14,15,16,17,18,19,20,21,22,23,24,25,26,27,28,29,30,31,32,33]. These include nomograms for the left ventricle (LV) [2,4,7,8,9,10,11,12,13,14,15,16,17], the right ventricle (RV) [10,17,30,31,32], the and atria [5,6,18,19,20,21]. Nonetheless, normal pediatric values for STE analysis remain elusive, necessitating thorough, time-intensive research. The largest online platforms for normal pediatric echocardiographic values do not incorporate strain values [24,25]. Only one online z-score platform [22] and a mobile application [23] have made available limited pediatric standard strain data [15,16,17]. Variations among vendors regarding STE values are well-established; however, the necessity for vendor-specific nomograms and the interchangeability of normal values calculated using different platforms remain uncertain [26,27,28,29]. While strain values generated by different vendors may vary, variations within software from the same vendor can also introduce significant discrepancies [26,27,28,29]. Advancements in technology have led to notable enhancements in the calculation of STE values [26,27,28,29]. Consequently, strain values produced using upgraded software versions may significantly differ from those calculated with prior versions [26,27,28,29]. Moreover, atrial strain analysis was initially executed using software originally designed for ventricular analysis [19,20,21], while, more recently, software explicitly tailored to atrial analysis has been introduced [18].

The present investigation aims to provide a review and update on major echocardiographic nomograms for speckle-tracking echocardiography.

## 2. Literature Search Criteria

In October 2024, a review of research was conducted utilizing three medical search engines: the National Library of Medicine, Science Direct, and the Cochrane Library. This review focused on Medical Subject Headings (MeSH) and the free-text terms “echocardiography”, “speckle tracking echocardiography”, and “normal values in children”.

The search parameters were further refined by incorporating the keywords “nomograms”, “z-scores”, and “healthy children”. Additionally, we identified other potentially relevant publications through a manual examination of references from all eligible studies and review articles, as well as the Science Citation Index Expanded available on Web of Science. The titles and abstracts of all articles identified through this search strategy were thoroughly evaluated. Manuscripts were excluded if they (a) utilized imaging techniques that differed from echocardiography, (b) contained a mixed population of adults and children, (c) evaluated fewer than 50 healthy children, or (d) were written in a language other than English.

The review was executed per the PRISMA 2020 statement [30]. All articles were evaluated independently by two specialists in pediatric echocardiography (M.C. and P.M.), and they were included in the study after reaching a consensus.

## 3. Results

### 3.1. Search Results

**Figure 1:**
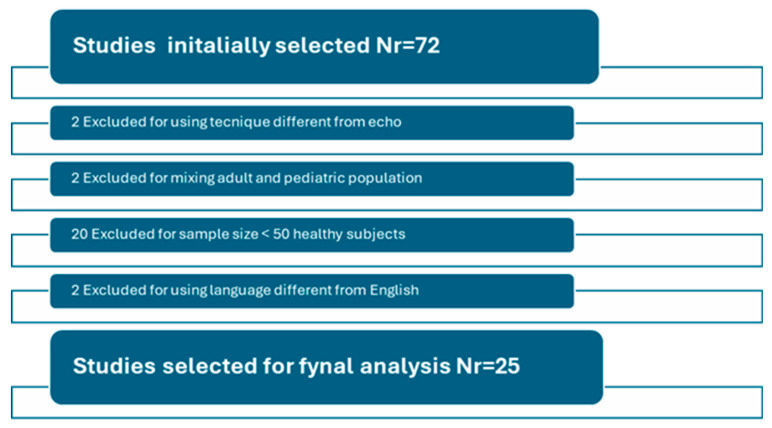
Selection diagram according to PRISMA guidelines.

Out of the 72 publications identified for potential inclusion in the study, 47 (63%) were excluded based on the above criteria, while 25 (27%) were ultimately selected for analysis and systematic review. Refer to Figure 1 and Table 1 and Table 2 for additional information.

In the initial section of this article, we will provide a general overview of the physics and basic principles of STE; then, we will provide a detailed examination of ventricular strain nomograms. In the subsequent section, the analysis will focus on atrial strain nomograms.

#### Physics and Basic Principles of STE

Strain is the deformation that occurs when a force is applied; specifically, myocardial strain represents the percentage change in myocardial length from a relaxed to a contractile state [31,32,33]. It can be negative (shortening or thinning) or positive (lengthening or thickening) relative to the initial end-diastolic length (L0) and is expressed as a dimensionless value, either as a fraction or a percentage [31,32,33]. *Lagrangian strain*, the most commonly used in cardiology, describes myocardial motion through space and time [31,32,33]. When deformation is related to the length at a specific moment (dt), the reference value changes during the process, and this is referred to as instantaneous natural strain. *Strain rate (SR)*, on the other hand, is defined as the temporal derivative of strain and is measured in s^−1^, being mathematically related to both tissue velocity and strain [31,32,33]. Speckle-tracking echocardiography utilizes speckles, which are small, stable acoustic markers in two-dimensional (2D) images generated by ultrasound backscatter in myocardial tissue [31,32,33]. These speckles result from the interaction between the ultrasound beam and the myocardium and change throughout the cardiac cycle due to the deformation of fibers, sheets, and collagen [31,32,33]. Because this deformation is relatively slow and coherent, speckle patterns change gradually and can be tracked over multiple cardiac cycles [31,32,33]. The main components of myocardial contraction include the following. (i) *Longitudinal*: movement from the base to the apex. (ii) *Radial*: thickening and thinning. iii) *Circumferential*: changes in length along the circular perimeter (short axis) [31,32,33]. The most validated clinical parameter in STE is *global longitudinal strain (GLS)*, which reflects the global longitudinal contraction of the myocardium, calculated from three different apical long-axis projections (four-chamber, three-chamber, and two-chamber views) [31,32,33].

### 3.2. Ventricular Strain

#### 3.2.1. Relationship of Strain Parameters with Age and Body Size Parameters

A mild negative correlation between ventricular strain parameters and age has been documented by various authors [2,3,7,8,9,10,12,14,18], with correlation coefficients ranging from 0.8 to 0.007. Notably, the relationships between strain parameters and age were not linear in two of the most comprehensive studies available [16,27]. In a substantial study involving 721 healthy children aged between 31 days and 18 years [16], LV GLS values were significantly higher in toddlers and infants (31 days to 24 months) compared to all other groups (*p* < 0.001), while considerably lower values were observed in older children (11–18 years) when juxtaposed with all age subgroups (*p* < 0.001). For left ventricular circumferential strain (CS), the distinctions among age groups were minimal, with older children (11–18 years) presenting higher medial and global strain values [16]. No significant differences among age groups were observed concerning RV GLS [16]. In another study comprising 1023 subjects under age 21 [27], a nonlinear relationship between mean strain and age was also reported, indicating that LV longitudinal strain (LS) and LV CS increase with age, peaking at 5 years before declining thereafter. Conversely, RV LS increased with age, peaking at 4 years and declining with age [27].

Mild negative correlations were identified between ventricular strain parameters and body surface area (BSA), with R values ranging from 0.34 to 0.002 [2,7,10,12,15,16].

#### 3.2.2. Gender

Most authors did not observe any gender differences [1,2,8,10,27]. Only one study [10] indicated that higher LV and RV LS values were present in females compared to males; however, these differences were minimal (*p* values ranging from 0.45 to 0.62). Additionally, Romanoviz et al. [27] reported elevated LV LS values in females, but this was confined to the 14- to 18-year-old age group and was interpreted primarily as differences associated with the varying timings of puberty between the sexes.

#### 3.2.3. Data Normalization and Expression

Most authors normalized their data by age groups and presented them as mean values, accompanied by the standard deviation [2,8,9,10,11,12,18,19,21,27]. A limited number of authors utilized z-scores [4,15,20,27], whereas percentiles were employed in only one instance [1]. In cases in which z-scores were applied [4,15,20,27], age was utilized for normalization [4,20,27], with only one study using BSA for this purpose [15].

Table 3 and Table 4 present the mean values of LV and RV strains categorized by major pediatric nomograms across various age groups.

#### 3.2.4. Comparison Among Vendors

- Inter-vendor variability

Several studies have evaluated the agreement among strain values generated by software from different vendors [26,28,29]. As presented in Table 5, good to excellent agreement was observed between the General Electric (GE Healthcare, Milwaukee, WI, USA) and TomTec platforms (TomTec Imaging Systems GmbH, Unterschleissheim, Germany), irrespective of the layer of analysis or the image format [26]. The agreement for LV GLS was found to be more robust, with an intraclass correlation coefficient (ICC) ranging from 0.88 to 0.9, a bias of 0.9 to 1.6, and a standard deviation (SD) between 1.9 and 2.3. In comparison, the ICC for global circumferential strain (GCS) was lower, ranging from 0.75 to 0.82, with a bias from 2.5 to 4.2 and an SD between 4.1 and 4.8 [26]. Disparities among vendors were more pronounced for children under 3 years, with GLS ICCs ranging from 0.62 to 0.92 as opposed to 0.9 to 0.95 for those older than 3 years; for GLC ICC, the ranges were 0.67 to 0.94 compared to 0.78 to 0.86 for children over 3 years [26]. Another study [29] compared different Philips (Philips Medical Systems, Best, The Netherlands) QLAB versions (10.5 and 10.8) and TomTec software 2D CPA 1.2.2 across three groups of children, including healthy subjects (*n* = 36), patients with ventricular paced rhythm (*n* = 36), and children exhibiting a flattened ventricular septum due to right ventricular pressure or volume load lesions (*n* = 36) [29]. Significant differences emerged between QLAB version 10.5 and all other software packages [27,29]. Conversely, good agreement was reported between QLAB version 10 and TomTec. TomTec’s low and high frame rate strain values differed only in children with a flattened septum due to right ventricular overload [26,29]. The only study comparing the GE and Philips platforms QLAB version 10 was conducted on 156 healthy children aged 1 month to 16.8 years, revealing a low level of agreement between these two platforms, with ICC values of 0.34 for longitudinal strain (lLS) and 0.12 for circumferential strain (CS) [28].

The differences among various software versions of the same vendor have also been analyzed [27,29]. A study evaluating these differences among subsequent versions of Philips software, specifically QLAB 10.8, AutoSTRAIN, and QLAB 10.5, revealed that the QLAB 10.5 platform consistently generated the highest strain values. In contrast, the QLAB 10.8 platform exhibited the lowest values [27,29]. For left ventricular strain (LS), differences were identified across age groups among the three platforms in all categories (*p* < 0.001), except for neonates and infants [27]. In neonates and infants, the mean strain values measured using QLAB 10.8 and AutoSTRAIN were found to be equivalent (neonates, mean difference = 0.004, SE = 0.005, *p* = 0.395; infants, mean difference = 0.006, SE = 0.004, *p* = 0.151); however, differences were evident when comparing QLAB 10.5 (*p* < 0.001 for all comparisons) [27]. Regarding LV CS, notable differences among age groups were observed across the three platforms for all age categories (*p* < 0.001), with higher strain values noted among individuals aged 1 to 10 years [27]. Strain values calculated using QLAB 10.8 were, on average, higher than those from QLAB 10.5 for all age groups except neonates [27]. For RV A4C LS, strain values from QLAB 10.8 were, on average, higher than those from AutoSTRAIN across all age groups, excluding neonates [27].

- Inter- and intra-sonographer variability

In the context of the Philips QLAB 10.8 [27,29], the intra-observer variability for left ventricular longitudinal strain (LV LS) was observed to be moderate, with an intraclass correlation coefficient (ICC) ranging from 0.5 to 0.79. Conversely, the inter-observer agreement was categorized as mild to good, with an ICC ranging from 0.8 to 0.96 [27,28,29]. In the case of QLAB Auto Strain, the intra-observer variability for LV LS was excellent, with an ICC exceeding 0.9. Meanwhile, the variability for RV LS was assessed as moderate to good, with an ICC of 0.78 for the RV free wall LS and an ICC of 0.62 for RV global LS [27]. The inter-observer variability for all measurements was also classified as excellent, with an ICC of no less than 0.8 [27]. Additionally, data obtained from GE and TomTec Echopac [26] demonstrated good intra-reader agreement for both LV LS and LV CS, with ICC values ranging from 0.85 to 0.96. The inter-observer agreement for LV GLS was categorized as good (>0.88), while the agreement for LV CS was found to be moderate (0.65–0.85) [26]. Furthermore, utilizing TomTec software 2D CPA 1.2.2, the intra-observer and inter-observer variability for LV LS based on images captured at a high frame rate was assessed as good, with ICC values of 0.82 and 0.92, respectively. However, the variability was moderate for pictures at a low frame rate, with ICC values of 0.65 and 0.8, respectively [29].

Major ranges of normality for LV and RV STE strain in the pediatric age group are reported in Table 4 and Table 5.

## 4. Atrial Strain

### 4.1. Maturational Variation

Significant variations in maturation were observed in atrial strain values [18,19,20,21]. Nomograms derived from two-dimensional (2D) STE illustrated a nonlinear positive correlation between left atrial (LA) reservoir strain (Sr) and age, alongside a nonlinear negative correlation of LA contractile strain with age, with rapid alterations observed during infancy [18,19,20]. Both LA and right atrial (RA) conduit strain values were diminished at younger ages, while contractile epsilon (ε) values were observed to be elevated at these earlier life stages [18]. In nomograms generated through three-dimensional (3D) STE, a decrease in all components of LA strain with advancing age was noted. However, the correlations of strain parameters with age were weak (r = 0.14 for global longitudinal strain and r = 0.31 for global three-dimensional strain) [20]. Interestingly, aside from age, no confounding variables, such as weight, height, BSA, and heart rate, exerted a significant influence on strain parameters [18,19].

### 4.2. Comparison Between P- and R-Gating and Ventricular-Specific and Atrial-Specific Software

Significant differences were observed in P-gating values, which were lower than R-gating strain values [18] for both the left LA and RA, with *p*-values less than 0.001 across all age groups [18]. Notable differences emerged between atrial strain values derived from atrial-specific software (QLAB 10) and those obtained using the previous ventricular-specific version (QLAB 9), adapted for atrial strain analysis. Atrial strain reservoir (Sr) values obtained through QLAB 10 atrial-specific software were generally lower than those acquired with QLAB 9. The LASr values calculated using QLAB 10 atrial-specific software were notably lower than those from QLAB 9 for the entire population (*p* < 0.001). In contrast, RAsr values were lower only in age groups 3 and 4 (*p* = 0.003) [18,19]. Furthermore, atrial strain conduit (Sct) values displayed differences between QLAB 10 and QLAB 9 [18,19]. Specifically, LASct values obtained with QLAB 10 were higher in younger patients (*p* = 0.03) but lower in older children (*p* = 0.003), while RA contractile strain (Sct) values were elevated across all age groups (*p* < 0.001) [18,19].

Table 6 presents the mean values of LA and RA strain categorized by age groups, as outlined by major pediatric nomograms.

## 5. Discussion

This review presents an updated overview of current pediatric nomograms for strain echocardiography (STE), emphasizing the availability of normal values for all significant strain parameters [1,2,3,4,5,6,7,8,9,10,11,12,13,14,15,16,17,18,19,20,21]. These values were calculated using various vendors and platforms, including the most recent updates from the same vendor [18,19,26,27,28,29]. However, data regarding right ventricular [16,18,27] and right atrial strain parameters [18,19,20] are limited. Most of the nomograms originate from Europe [7,10,14,16] and North America [1,2,4,13,15,17,21,27], with a smaller number from Asia [3,8] and North Africa [5,9].

In all conducted studies, the correlations between STE strain parameters and age and body size parameters were found to be weak [2,8,9,10,11,12,18,19,21,27]. The decision to utilize age rather than BSA for data normalization was made due to the observation that, despite being weak, the correlations of strain parameters with age were more substantial than those observed with BSA [2,8,9,10,11,12,18,19,21,27]. However, these weak (often non-linear) correlations between strain parameters and age and body size parameters hindered the ability to compute Z-scores with adequate statistical power [16,18,20]. Consequently, the data have predominantly been presented as mean values accompanied by standard deviation. It is noteworthy that the formulation of Z-score equations exhibiting low R^2^ values may amplify the variability observed between the upper and lower extremities within the range of normality. In numerous studies [15,16,18,20], the relationships between strain parameters and BSA were exceedingly weak (approximately zero); hence, a precise estimation of the population mean is not feasible, thereby obstructing the calculation of Z-scores with sufficient statistical power [16,18,20]. Z-scores calculated with R^2^ values in the vicinity of zero may result in upper and lower limits of normality exhibiting more than a 20-point discrepancy among the “observed” lower and upper limits of normality. The feasibility of smoothed percentiles has also been explored [16,18,20]; however, similarly to Z-scores, a robust relationship among echocardiographic parameters is requisite to generate a percentile chart. When Z-scores have been employed [3,15,27], either low R^2^ values have been accepted (e.g., ranging from 0.002 to 0.046) [15] or details about the accuracy of the Z-score equations have not been provided [3,27].

A significant consideration in STE analysis pertains to the discrepancies arising from utilizing software provided by various vendors [26,27,28,29]. The inter-vendor variability between GE and TomTec was satisfactory to excellent [26], while the comparability between Qlab10 and TomTec was assessed as good [29]. In contrast, the limited data available concerning the GE and Philips platform QLAB 10 [28] exhibited a low level of agreement between these two platforms. Comparisons of different software from the same vendor [27] indicated a good concordance between QLAB 10 and Autostrain, particularly for neonates and infants [27]. However, significant disparities were noted for older software versions (QLAB 5) [27]. Furthermore, infra-operator and inter-operator variabilities within the same software demonstrated good consistency [16,26,27,29], with a few exceptions noted [28].

The data available regarding atrial strain are notably limited and diverse. Recent investigations [3,18] have utilized software developed explicitly for atrial analysis, in contrast to earlier nomograms [19,20,21] that employed software initially designed for ventricular assessment but adapted for atrial evaluation. The findings derived from the newly created atrial-specific software indicated significant discrepancies compared to those obtained through the ventricular software repurposed for atrial study [3,18]. Therefore, similar to practices established for adults [34,35], it is imperative in a pediatric context to ensure that comparisons are conducted using data from identical software and methodologies to facilitate effective clinical monitoring of patients.

Several studies [8,18,20] have provided pediatric normative values for 3D STE analysis, indicating similar age-related variations and trends, as observed with 2D STE. For the left ventricle, the mean GLS values reported using 3D STE are somewhat lower compared to those documented for 2D STE (3D GLS mean values ranged from −17.31 to –22.8 versus 26.0 to 24.00) [8,16,18]. The three-dimensional left ventricular circumferential strain values are also lower than those obtained with 2D STE [18]. For atrial strain values derived from 3D STE [20], mean values have not been delineated, complicating comparisons with 2D STE results [18,19,20]. Theoretically, 3D techniques should provide advantages over 2D methods by eliminating out-of-plane artifacts and enabling the calculation of the circumferential component of left atrial strain. In research conducted by Ghelani and colleagues [20], the circumferential component of 3D left atrial strain was identified as a significant contributor to most age-related changes.

### 5.1. Strengths and Limitations

This study possesses several strengths. It represents an inaugural investigation to deliver a comprehensive overview of pediatric normative values concerning all major strain parameters. Furthermore, it includes a critical analysis of the variance among software from different vendors and software versions from the same vendor. However, this research is not without its limitations. The heterogeneity of the data inhibited the capacity to conduct a meta-analysis. The varying methods for normalizing data and the presentation of normalized data additionally complicate comparisons of the ranges of normality proposed by different authors. Comparisons among various vendors are constrained [27,28,29], and extensive studies comparing platforms from other vendors are recommended. Notably, the differences in STE values among children of diverse racial and ethnic groups have yet to be explored. Most studies have predominantly concentrated on longitudinal strain [4,5,8,10,15,18,19,21]. Currently, the strain methodology is recommended in adult guidelines [36].

### 5.2. Practical Applications and Future Directions

The results of this review underscore several practical applications of STE in the field of pediatric cardiology. The nomograms provided for strain parameters represent a significant resource for clinicians, thereby facilitating the assessment of ventricular and atrial function in pediatric patients suffering from congenital and acquired heart diseases. By functioning as a reference framework, these nomograms permit the early detection of abnormal strain values and promote standardized diagnostic and monitoring practices. The integration of these normative values into routine clinical workflows has the potential to enhance diagnostic accuracy and improve patient outcomes. Nonetheless, practitioners must remain vigilant regarding the variability among vendors and software versions, ensuring the consistent application of methodologies and the meticulous interpretation of strain measurements.

In addition to pragmatic considerations, this review highlights considerable deficiencies within the existing research landscape that necessitate further inquiry. Despite the presence of normative data pertaining to left ventricular strain, the scarcity of information regarding right ventricular and atrial strain parameters constitutes a significant void.

Moreover, the frail and nonlinear relationships observed between strain values, age, and body surface area impede the formulation of robust Z-score equations, thereby constraining the statistical efficacy of the current nomograms.

Future research endeavors should concentrate on addressing the aforementioned limitations. It is imperative to conduct comprehensive studies aimed at augmenting the dataset concerning right ventricular and atrial strain parameters, which are presently underrepresented. Right ventricular strain is gaining increasing recognition in the assessment of children with congenital heart disease (CHD) [37,38,39]. Speckle-tracking echocardiography (STE)-derived global longitudinal strain (GLS) and free wall longitudinal strain (LS) are becoming essential tools for evaluating right ventricular function in children with both congenital and acquired CHDs, as well as in those with pulmonary hypertension of various etiologies [37,38,39]. In a normal right ventricle, longitudinal systolic function is the primary determinant of overall RV systolic performance [37,38,39]. However, in congenital heart disease—particularly in conotruncal defects—RV failure can result from multiple factors following surgical and/or percutaneous correction [37,38,39]. These include pressure or volume overload, electromechanical dyssynchrony, increased myocardial fibrosis, impaired coronary perfusion, restricted diastolic filling capacity, and adverse ventricular interactions between the left (LV) and right ventricle (RV) [37,38,39]. Moreover, STE is increasingly utilized for the evaluation of the systemic right ventricle, further expanding its role in the comprehensive assessment of RV function in CHD [39]. Understanding atrial deformation by STE may also be crucial in congenital heart disease and cardiomyopathies, particularly in the pediatric population, where there is an unmet need to detect subclinical left myocardial dysfunction before overt heart failure develops [40].

Furthermore, standardizing strain measurements across various vendors and software platforms is essential for enhancing inter-vendor reliability and ensuring the widespread applicability of STE within clinical practice. Additionally, a more in-depth exploration of potential demographic and ethnic variability in strain parameters is vital, given that existing studies have primarily focused on homogenous populations. Lastly, the formulation of pediatric-specific software, particularly for atrial strain analysis, is necessary to enhance measurement precision and clinical utility.

By addressing these deficiencies, the clinical utility of Strain Tissue Elasticity (STE) can be significantly enhanced, thereby facilitating more accurate, consistent, and comprehensive evaluations in pediatric patients. Ongoing advancements in research and standardization will further solidify the role of STE as an indispensable instrument in the field of pediatric cardiology.

## 6. Conclusions

We offer a comprehensive review and current updates on pediatric nomograms for STE, which may assist clinicians in interpreting pediatric STE values. To interpret STE values accurately in the pediatric population, it is crucial to recognize the similarities and differences among various technologies and utilize appropriate nomogram sources. Given the inter-vendor and intra-vendor discrepancies, it is advisable to compare data from the same software and, for atrial assessments, employ identical methods (e.g., P-gated or R-gated).

## Figures and Tables

**Table 1:** Major pediatric STE nomograms for ventricular strain values.

Author	Population	Measures	Software	Data Norm	Data Expression
Adar A 2019 USA [2]	*n* = 312 3 days–20.5 years	LV LS, CS, and synchrony	Echo: Philips Epiq Software: QLAB v.10.5 (Philips)	Age groups	Mean, SD
Harrington JK 2021 USA [4]	*n* = 577 1–18 years	LV SR	Echo: Philips Software: QLAB Version 5 (Philips)	Age	Z-scores
Koopman LP 2019 The Netherlands [7]	*n* = 103 Mean 10.8 years IQR 7.3–14.3 years	LV LS, CS	Echo: Philips IE33 Software: QLAB 9.0 (Philips)	Age groups	Mean, SD Percentiles
Romanowicz J 2023 USA [27]	*n* = 1032 <21 years old	LV and RV LS, LV CS	Echo: Philips Epiq Software: Autostrain, QLab 10.5 10.8	Age	Mean, SD Z-scores
Kamel H 2022 Egypt [9]	*n* = 200 3.832 ± 1.522 years Range 0.1–5.9 years	LV GLS, GCS, GRS 2D and 3D	Echo: Vivid E9 (GE) Software: EchoPAC V113 (GE)	Age groups	Mean, SD
Kotby AA 2023, Egypt [5]	*n* = 250 1–16 years	LV LS	Echo: GE Software: EchoPAC v206	Age groups	Mean, SD
Aristizábal-Duque CH 2022 Spain [10]	*n* = 156 6–17 years	LVGLS, RVGLS, RV free wall LS, LA	Echo: Philips IE33 Software: 13.0 of Qlab 13 (Philips) °	Age groups, BSA	Mean, SD
Klistisic L, 2013 The Netherlands [14]	*n* = 183 0–19 years	LV LS, CS, RS	Echo: Vivid 7 GE Software: EchoPAC GE v206	Age groups	Mean, SD
Marcus K, 2011 USA [13]	*n* = 144 0–19 years	LV LS, CS, RS	Echo: Vivid 7 GE Software: EchoPAC GE v206	Age groups	Mean, SD
Zhang L, 2013 China [8]	*n* = 226 0–18 years	LV 3D STE LS, CS, RS	Echo: Philips IE33 Software: Tomtec 4D Cardio-View 3.0	Age groups	Mean, SD
Cantinotti M, 2018 Italy [16]	*n* = 721 31 days–18 years	LV, LS, CS RV LS	Echo: Epiq/IE33 (Philips) Software: QLAB 9 Philips	Age groups, gender	Mean, SD
Dallaire F, 2016 Canada [15]	*n* = 233 1–18 years	LV LS, CS	Echo: Vivid 7 GE Software: EchoPAC GE 7	BSA	Z-scores
Acheampong B, 2023 USA [1]	*n* = 142 0–18 years	LV LS, CS, RS	Echo: Philips and Siemens Software: Cardiac Performance Analysis version 3.0 *	Age groups	Percentiles

CS = circumferential strain, GCS = global circumferential strain, IQR = interquartile range, LA = left atrium, LV = left ventricle, LS = longitudinal strain, *n* = number, GLS = global longitudinal strain, GRS = global radial strain, RV = right ventricle, RS = radial strain, SR = strain rate, GE = General Electric Ultrasound, Horten, Norway, TomTec = TomTec Imaging Systems, Germany, Siemens Healthineers Erlangen, Germany; ° which integrates the TOMTEC auto-strain software; * independent software.

**Table 2:** Major pediatric STE nomograms for atrial strain values.

Author	Population	Measures	Software	Data Norm	Data Expression
Cantinotti M, Italy [18,19]	*n* = 836 31 days–18 years	2D LA and RA strain	Echo: Epiq/IE33 (Philips) Software: QLAB) and QLAB 10 ° (Philips)	Age groups	Mean, SD
Ghelani S, 2013 USA [20]	*n* = 196 4 days–20.9 years	3D LA volumes and strain	Echo: Philips IE33 Software: 4D LV Analysis, Tomtec 3.1	Age	Z-scores
Kutty S 2013, USA [21]	*n* = 153 3–20 years	2D LA and RA strain	Echo: GE Vivid 7, Software: EchoPAC Bt11 GE	Age groups	Mean, SD
Jimbo S 2020 Japan [3]	*n* = 112 (median 12.0 years; range 6–16 years)	2D LA strain and SR	Echo: NR Software: Tomtec 2D CPA 1.2.2	Age groups	Z-scores
Aristizábal-Duque CH 2022 Spain [10]	*n* = 156 6–17 years	LA	Echo: Philips IE33 Software: 13.0 of QLAB 13 (Philips) °	Age groups, BSA	Mean, SD

LA = left atrium, *n* = number, NR = not reported, RA = right atrium, SD = standard deviation, SR = strain rate, 2D = two-dimensional, 3D = three dimensional, TomTec = TomTec Imaging Systems, Germany, GE = General Electric Ultrasound, Horten, Norway, ° dedicated to atria.

**Table 3:** Major range of normality proposed by major nomograms for LV strain.

	Neonates	Infants	1–5 Years	5–10 Years	10–14 Years	14–18 Years	18–21 Years
Romanowicz J [27] LV GLS							
QLAB 8	−22 ± 3	−23 ± 1	−23 ± 2	−23 ± 3	−22 ± 3	−21 ± 3	−21 ± 4
Autostrain	−21 ± 1	−23 ± 3	−25 ± 3	−25 ± 3	−24 ± 2	−22 ± 3	−23 ± 3
LV GCS QLAB 8	−27 ± 4	−29 ± 4	−32 ± 4	−31 ± 3	−30 ± 4	−30 ± 4	−29 ± 4
Kamel H [9] GE EchoPAC	<1 year	>1≤2 years	>2≤3 years	>3≤4 years	>4≤5 years	>5≤6 years	
LV GLS 2D	−23.3 ± 7.3	−24.5 ± 1.1	−23.5 ± 0.5	−22.3 ± 0.7	−21.1 ± 0.7	−19.9 ± 0.9	
LV GCS 2D	−18.9 ± 0.9	−19.1 ± 0.7	−19.4 ± 1.3	−18.7 ± 1.2	−18.9 ± 0.9	−19.0 ± 1.58	
LV GRS 2D	48.4 ± 2.7	44.5 ± 1.1	43.4 ± 0.8	42.4 ± 0.8	41.3 ± 0.8	40.0 ± 1.1	
LV GLS 3D		−22.8 ± 2.8	−20.9 ± 0.8	−20.1 ± 0.8	19.2 ± 0.9		
LV GCS 3D		−14.6 ± 1.6	−14 ± 2.2	−13.7 ± 1.8	−13.9 ± 2.4	−13.9 ± 2.1	
LV GRS 3D		50.4 ± 4.5	47.7 ± 2.1	46.8 ± 1.1	45.6 ± 1.5	47.21 ± 2.3	
Aristizábal-Duque [10] QLAB 13				6–9 years	10–12 years	12–17 years	
LV GLS				−27.3 ± 2.1	−26.2 ± 2.7	−23.3 ± 2.25	
Klistisic L [14]		<1 year	1–4 years	5–9 years	10–14 years	15–19 years	
LV GLS GE EchoPAC		−20.6 ± 3.1	−23.6 ± 1.5	−23.1 ± 2.2	−21.8 ± 2.1	−20.8 ± 1.8	
Zhang L [8] 3DSTE Philips/Tomtec		<1 year	1–5 years	5–9 years	9–13 years	13–18 years	
GLS		−17.3 ± 2.3	−17.3 ± 2.6	−17.6 ± 2.2	−18.7 ± 1.7	−16.6 ± 2.8	
GCS		−16.8 ± 2.7	−17.1 ± 3.2	−17.6 ± 3.4	−18.8 ± 3.3	−16.3 ± 4.1	
GRS		61.56 ± 18.8	60.3 ± 13.4	59.5 ± 13.3	64.6 ± 8.4	56.6 ± 14.9	
GS		−29.7 ± 4.5	−30.1 ± 3.5	−30.2 ± 3.6	−31.5 ± 2.6	−28.9 ± 4.5	
Cantinotti M [16] QLAB 8		31 days–24 months	2–5 years	5–11 years		11–18 years	
GLS		−26.0 ± 2.3	−25.0 ± 2.2	−24.7 ± 2.3		−24.0 ± 2.3	
GCS		−24.6 ± 4.2	−23.3 ± 4.3	−24.5 ± 4.5		−25.4 ± 4.2	

GE = General Electric Ultrasound, Horten, Norway, LV = left ventricle, GCS = global circumferential strain, GLS = global longitudinal strain, GRS = global radial strain, 2D = two-dimensional, 3D = three-dimensional, QLAB = software of Philips Medical Systems, Best, The Netherlands, TomTec = TomTec Imaging Systems, Germany.

**Table 4:** Major range of normality proposed by major nomograms for RV longitudinal strain.

Romanowicz J [27]	Neonates	Infants	1–5 Years	5–10 Years	10–14 Years	14–18 Years	18–21 Years
RV GLS							
QLAB 8	−24 ± 3	−28 ± 5	−30 ± 4	−28 ± 4	−27 ± 5	−26 ± 3	−27 ± 5
Autostrain	−22 ± 2	−24 ± 4	−28 ± 3	−26 ± 3	−25 ± 4	−23 ± 3	−23 ± 4
RV free wall							
Autostrain	−25 ± 2	−29 ± 5	−32 ± 5	−30 ± 4	−29 ± 5	−27 ± 4	−27 ± 4
Aristizábal-Duque [10] QLAB 13				6–9 years	10–12 years	12–17 years	
RV GLS				28.7 ± 3.2	26.4 ± 4.2	24.9 ± 3.7	
RV free wall				32.6 ± 3.7	29.8 ± 4.3	28.3 ± 4.1	
Cantinotti M [16]		31 days to 24 months	2–5 years	5–11 years		11–18 years	
RVGLS		−25.4 ± 3.9	−25.9 ± 4.0	−25.8 ± 4.7		−25.0 ± 4.1	

RV = right ventricle, GLS = global longitudinal strain, QLAB = software of Philips Medical Systems, Best, The Netherlands.

**Table 5:** Major pediatric studies comparing STE values among different platforms.

Author	Population	Measure	Software
Amedro P 2019 France [28]	Nr = 156 healthy 1 month–16.8 years.	LV and RV	GE (EchoPAC version 112) and Philips (QLAB 10)
Ramlogan S, 2019 USA [26]	38 healthy 15 cardiomyopathies 1–18 years	LV LS, CS	GE EchoPAC and TomTec software 2D CPA 1.2.2, and TomTec at compressed rate
Romanowicz J 2023 USA [27]	Nr = 1032 healthy <21 years old	LV and RV LS, LV CS	Autostrain QLAB 8 and QLAB 5
Ferraro A, 2020 USA [29]	Nr = 108 Group 1: healthy Group 2: ventricular paced rhythm Group 3: flattened IVS	LV LS, CS	QLAB (versions 10.5 and 10.8) Philips and TomTec

CS = circumferential strain, GE = General Electric Ultrasound, Horten, Norway, IVS = interventricular septum, LV = left ventricle, LS = longitudinal strain, RV = right ventricular, QLAB = software of Philips Medical Systems, Best, The Netherlands, TomTec = TomTec Imaging Systems, Germany.

**Table 6:** Major range of normality proposed by major nomograms for atrial longitudinal strain.

Aristizábal-Duque CH 2022 Spain [10] QLAB 13				6–9 yrs	10–12 yrs	10–17 yrs
LASr				60.2 ± 9.4	57.6 ± 12.3	54.8 ± 9.5
LAScd				47.3 ± 8.9	43.9 ± 10.4	42.7 ± 8.9
LASct				13 ± 5.4	13.7 ± 5.6	12 ± 5.3
Cantinotti M [18,19] QLAB Atrial Software Version 5		31 d to 24 mths	2–5 yrs	5–11 yrs		11–18 yrs
RR LASr		49.3 ± 15.0	54.2 ± 16.4	53.2 ± 15.7		50.5 ± 16.5
PP LASr		41.3 ± 10.9	47.4 ± 13.6	46.2 ± 13.2		44.4 ± 13.2
RR LAScd		31.5 ± 11.3	40.0 ± 14.0	39.5 ± 14.3		37.5 ± 13.5
PP LAScd		26.8 ± 9.7	35.2 ± 12.5	34.9 ± 12.9		33.4 ± 12.0
RR LASct		18.2 ± 9.4	14.3 ± 6.7	14.1 ± 6.2		13.2 ± 7.1
PP LASct		14.9 ± 6.3	12.2 ± 4.9	12.2 ± 4.7		11.4 ± 5.6
RR RASr		45.3 ± 16.4	49.1 ± 16.2	47.9 ± 17.2		48.0 ± 16.5
PP RASr		37.4 ± 11.8	41.5 ± 13.0	41.1 ± 13.8		41.5 ± 13.4
RR RAScd		25.2 ± 11.0	31.6 ± 13.9	32.2 ± 14.1		33.2 ± 13.3
PP RAScd		21.2 ± 10.4	27.3 ± 12.9	28.1 ± 12.7		29.0 ± 11.5
RR RASct		21.0 ± 11.4	17.9 ± 8.2	16.1 ± 8.2		15.4 ± 7.2
PP RASct		16.8 ± 7.7	14.8 ± 5.9	13.5 ± 6.1		13.1 ± 5.6
QLAB Ventricular Software Version 5						
RR LASr conv meth		52.2 ± 9.3	55.1 ± 9.9	58.2 ± 10.2		57.2 ± 10.5
RR LASct conv meth		14.6 ± 6.5	12.2 ± 6.0	14.1 ± 7.2		15.1 ± 7.1
RR RASr conv met		46.1 ± 8.9	48.5 ± 10.5	50.9 ± 10.8		51.4 ± 10.7
RR RASct conv meth		11.7 ± 6.1	11.6 ± 5.7	11.9 ± 6.3		12.9 ± 5.4

PP = P-gated, RR = R-gated, LA = left atrium, RA = right atrium, Sr = reservoir strain, Scd = conduit strain, Sct = contractile strain, yrs = years, mths = months, QLAB = software of Philips Medical Systems, Best, The Netherlands.

## Data Availability

The data presented in this study are available upon request from the corresponding author.
